# Application of Single-Cell Approaches to Study Myeloproliferative Neoplasm Biology

**DOI:** 10.1016/j.hoc.2021.01.002

**Published:** 2021-04

**Authors:** Daniel Royston, Adam J. Mead, Bethan Psaila

**Affiliations:** a Nuffield Division of Clinical Laboratory Sciences, Radcliffe Department of Medicine and NIHR Biomedical Research Centre, University of Oxford, Headley Way, Oxford OX39DS, UK; b Medical Research Council (MRC) Molecular Haematology Unit, MRC Weatherall Institute of Molecular Medicine, NIHR Biomedical Research Centre, University of Oxford, Headley Way, Oxford OX3 9DS, UK

**Keywords:** Myeloproliferative neoplasm, Single-cell genomics, Digital pathology

## Abstract

Philadelphia-negative myeloproliferative neoplasms (MPNs) are an excellent tractable disease model of a number of aspects of human cancer biology, including genetic evolution, tissue-associated fibrosis, and cancer stem cells. In this review, we discuss recent insights into MPN biology gained from the application of a number of new single-cell technologies to study human disease, with a specific focus on single-cell genomics, single-cell transcriptomics, and digital pathology.

## Key points

• Myeloproliferative neoplasms (MPNs) are an excellent tractable disease model for the application of single-cell approaches to study human disease.
• Single-cell genetic analysis of MPNs has provided important insights into disease latency and the importance of the order of mutation acquisition during disease pathogenesis, of broader relevance for cancer biology.
• Single-cell transcriptomics has revealed aberrant megakaryocyte differentiation trajectories in persons with myeloproliferative neoplasms.
• Digital pathology analysis combined with deep learning allows objective analysis of megakaryocyte heterogeneity in persons with MPNs.

## Introduction

**Fig. 1:**
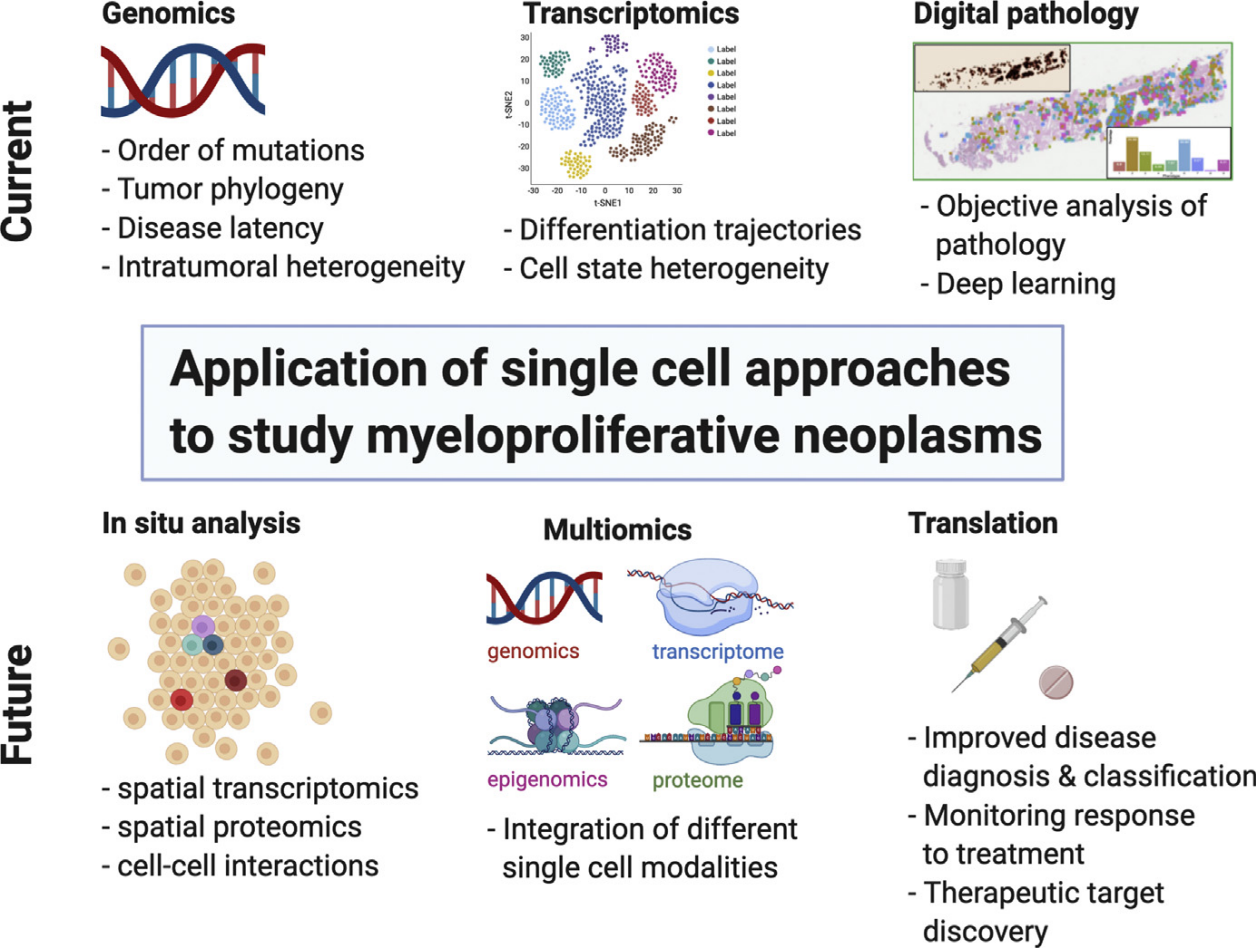
Overview of single-cell approaches used to study MPN: current methods and future applications. Created with BioRender.com.

Philadelphia-negative myeloproliferative neoplasms (MPNs) are an excellent tractable disease model of a number of aspects of human cancer biology, including genetic evolution, tissue-associated fibrosis, and cancer stem cells.^1^^,^^2^ MPN is associated with long disease duration, well-characterized normal hematopoietic hierarchy, ability to purify cell populations by flow cytometry, and ease of accessibility of tissue derived from the malignant clone, facilitating the study of how this disease perturbs normal blood cell development through time. Accordingly, new technologies developed to study cancer biology have often been pioneered for the study of MPN. In this review, we discuss recent insights into MPN biology gained from the application of a number of new single-cell technologies to study human disease: single-cell genomics, single-cell transcriptomics and digital pathology (Fig. 1).

## Single-cell genomics

In the era of personalized medicine, genetic intratumoral heterogeneity (ITH) is increasingly recognized as a critical factor defining the behavior of a particular tumor in terms of clinical presentation, response to treatment, and risk of disease progression.^3^ Although bulk next generation sequencing (NGS) techniques have undoubtedly provided extensive insights into genetic diversity of clones within any given tumor,^4^^,^^5^ ultimately ITH can only be fully resolved using single-cell technologies.^6^ For example, inferring clonal structures from bulk variant allele fractions is inherently confounded by the presence of loss of heterozygosity or convergent evolution in which the same genetic events might occur multiple times in the same tumor.^7^^,^^8^ This makes it difficult to elucidate which mutations are present in the same clone, to accurately measure clonal diversity during therapy, to track disease evolution, or determine the order of mutations.

MPN has proven to be an important disease model providing an illustration of how single-cell genomics techniques can be applied to provide new insights into disease biology and how the technology might be moved toward application for precision medicine. Indeed, to a degree, single-cell genomics approaches are already in routine clinical use in myeloid diseases through application of cytogenetic analysis, including fluorescent in situ hybridization. The most common driver mutation in MPN occurs in exon 14 of the Janus kinase 2 (*JAK2*) gene, *JAK2V617F*, causing constitutive JAK-signal transducer and activator of transcription (JAK-STAT) signaling and driving the aberrant proliferation that is characteristic of MPN. Subsequently other mutations causing aberrant JAK-STAT signaling in MPN have been reported to occur in patients with MPN who are negative for the *JAK2V617F* mutation,^9^, ^10^, ^11^ confirming that constitutive JAK-STAT signaling is the key pathway driving MPN phenotype.^12^ The mutational landscape of MPN has been extensive studied, revealing that MPNs are genetically relatively simple compared with other tumors.^12^^,^^13^ Together with other disease characteristics described in the introduction, this makes MPN an ideal tractable model for the application of single-cell genomics to understand the genetic evolution and clonal selection of mutant cells during disease development and progression. Consequently, single-cell assessment of genetic clonal evolution in MPN already has a rich history for the study of MPN over many years. Earlier studies benefited from the application of well-established clonogenic hematopoietic assays,^2^ overcoming limitations of direct genetic analysis of single cells by providing an expanded cell population derived from a single cell for genetic analysis. For example, through the analysis of X-chromosome linked polymorphisms, including the study of single-cell–derived hematopoietic colonies, it has long been appreciated that MPNs are clonal diseases^14^^,^^15^ that develop on acquisition of a disease-initiating mutation in a single multipotent hematopoietic stem cell (HSC).^1^ This mutant HSC clone undergoes a poorly understood process of clonal expansion over time, with subsequent proliferation of mature cells of the myeloid lineages.^1^ At the time that the *JAK2V617F* mutation was first described,^16^ it was demonstrated that *JAK2V617F* selectively promoted the growth of single-cell–derived erythropoietin-independent colonies in patients with polycythemia vera (PV).^17^ This approach was also used to characterize clonal diversity in patients with MPN with concurrent *JAK2V617F* mutation and a cytogenetic abnormality, demonstrating that these genetic events were present in the same clone in some patients but in separate clones in others.^18^ Furthermore, combined analysis of *JAK2V617F* zygosity and loss of heterozygosity breakpoints using microsatellite markers in 6495 colonies revealed that *JAK2V617F* homozygous clones are recurrently acquired in patients with PV and patients with essential thrombocythemia (ET).^19^ However, PV was typically associated with presence of a dominant homozygous subclone, unlike in ET. The study of single-cell–derived colonies in MPN has more recently provided novel insights into the importance of the order of acquisition of driver mutations. Analysis of colonies derived from single cells from patients with JAK2 and TET2 co-mutated MPN revealed differences in disease phenotype and response to targeted therapy that were dependent on the order of acquisition of these mutations.^20^ The acquisition of *JAK2V617F* first, followed by *TET2* mutation, was more likely to result in a PV phenotype, typically in younger patients, but if *JAK2V617F* was acquired on a *TET2*-mutated background (“*TET2*-first”), an ET phenotype was more frequent. Similar observations also applied to DNMT3A and JAK2 mutations.^21^ Together these studies nicely illustrate how the study of MPN can provide insights into pathways of genetic evolution of broader relevance for cancer biology.

Although analysis of hematopoietic colonies is a powerful approach, it is also associated with certain limitations and potential biases, as not all stem/progenitors are capable of growing colonies in vitro*.* Direct genetic analysis of single cells provides a potential solution, but is more challenging than analysis of material derived from colonies due to the very small amount of starting material and extensive amplification required.^22^ Single-cell mutation analysis can be carried out by targeted NGS of known mutations, single-cell exome, or whole-genome sequencing.^23^ A number of methods have been developed, each with advantages and also limitations in terms of the spectrum of mutations that can be analyzed and the sensitivity and specificity of mutation detection.^22^^,^^23^ Whole-genome and whole-exome techniques allow the characterization of new genetic events, whereas targeted techniques rely on detection of known mutations within a given tumor. Targeted mutational analysis is a higher throughput and more cost-effective approach than whole-exome or genome sequencing of single cells with improved sensitivity and specificity of mutation detection and high-throughput commercially available droplet-based platforms.^24^ Whole-genome single-cell techniques are typically associated with higher rates of allelic dropout, although new approaches to study low-input genomic DNA look set to change this.^6^^,^^25^ To help address such technical problems, a number of bioinformatics tools to resolve the tumor phylogenetic trees have been developed.^26^, ^27^, ^28^

One of the first examples of single-cell exome sequencing in human cancer was a case of JAK2-mutation negative MPN, where 58 cells were sequenced, revealing monoclonal evolution of the disease in this patient and potential new candidate mutations driving clonal evolution.^29^ The integration of single-cell genotyping with fluorescence-activated cell-sorting purification of specific cell populations allows mutations to be mapped to distinct phenotypically defined cell types, confirming that all mutations in MPN can be tracked back to the phenotypic HSC population.^30^ Single-cell targeted mutation analysis in serial samples from patients with myelofibrosis has revealed the very high level of clonal dominance in the CD34+ compartment seen in almost all patients with myelofibrosis.^31^ This study also elucidated pathways of clonal evolution during JAK2 inhibitor therapy, and demonstrated that complex clonal architecture correlates with risk of disease progression, particularly in association with the acquisition of RAS/RTK pathway mutations.^31^

New technologies now allow targeted mutational analysis of single cells to be done in a high-throughput manner using droplet-based approaches. For example, some of the first examples of direct single-cell sequencing using the Tapestri platform report the study of patients with MPN. In rare cases in which JAK2, CALR, and/or MPL mutations co-occur, this approach was used to determine that the mutations are present in independent clones.^32^ In a landmark study, the Tapestri platform has also recently been used to analyze 740,526 cells from 123 patients with myeloid malignancies, including cases of MPN that have progressed to secondary acute myeloid leukemia (AML).^24^ As might be expected, the number of mutations present and clonal diversity was higher in AML than MPN and also higher in MPN than in clonal hematopoiesis. In 4 of 6 patients with transformation of MPN to secondary AML, a new dominant subclone emerged that in some cases was present as a minor subclone during chronic phase. In this study, the investigators also describe an exciting new methodology to combine protein expression at the single-cell level with genotyping to link phenotype and genotype.

Perhaps the most remarkable recent finding, revealed through single-cell genomics approaches, relates to the origins and disease latency of MPN. These studies used state-of-the-art single-colony whole-genome sequencing lineage tracing approaches that rely on identification of background somatic mutations as a “molecular clock” to determine the timing of clonal expansion and disease development following acquisition of the *JAK2V617F* mutation.^33^^,^^34^ With the caveat that both papers are available only as “preprints” and have not yet undergone peer review, it is striking that both studies reach a similar conclusion that the *JAK2V617F* mutation was reported to be acquired typically decades before disease development; remarkably, in many cases, the mutation was acquired in utero or in early childhood and yet only caused disease after many decades in adult life. In the study from the Cambridge group, 448,553 somatic mutations were identified and used to determine clonal dynamics in 843 hematopoietic colonies from 10 patients with MPN. This study estimated the median latency between *JAK2V617F* acquisition and disease onset to be 31 years, with remarkable interpatient variation in fitness advantage of the MPN clone.^33^ The study by Van Egeren and colleagues^34^ analyzed a smaller number of colonies from 2 patients, also concluding that there was a disease latency of decades between *JAK2V617F* acquisition and disease onset. This long latency is particularly striking in view of the observation that many persons with normal hematopoietic parameters have evidence of a small *JAK2V617F* clone.^35^ This suggests that many persons acquire a *JAK2V617F* mutation and live with this mutation for decades, and perhaps in many cases lifelong, without ever developing disease. The challenge is now to understand the heterogeneity of clonal fitness advantage exerted by the *JAK2V617F* mutation in HSCs. It is likely that this will involve an interplay among germline genetics influencing HSC biology,^36^ heterogeneity of the HSC of origin, and extrinsic factors such as “inflammaging.”^37^ To unravel this crucial aspect of MPN biology will no doubt require extensive use of single-cell methodologies that look set to be at the forefront of MPN research in coming years.

## Single-cell transcriptomics

Single-cell RNA-sequencing (scRNAseq) is the most widely applied assay in single-cell genomics, and is extensively used to provide a comprehensive and unbiased assessment of normal cellular and molecular tissue architecture and their perturbations in disease states. Over the past decade, experiments have massively expanded in their scale and implementation, due to technological advances resulting in high-throughput methods that are relatively easy to implement.^38^^,^^39^ In parallel, there now exists a wealth of user-friendly and open-source computational pipelines for data analysis.^40^ Transcriptional profiling of cells individually has several advantages over “bulk” analyses, including detection of rare cell types; determination of whether differences between samples are due to differences in the frequencies of cell types present or alternatively changes in individual cell phenotype; and exploration of combinatorial patterns of gene expression and differentiation trajectories.

A typical analytical pipeline includes organizing cells according to their transcriptional profiles into discrete groups, or “clusters” that correlate with cell type or state.^40^ Although cells are captured as transcriptional “snapshots,” their differentiation trajectories can be inferred computationally using trajectory analyses or ordering over “pseudotime,” to identify key transition states and bifurcation points.^41^, ^42^, ^43^, ^44^ In addition, studying the ratio of spliced versus unspliced mRNA, or the “RNA velocity,” can be used to predict the future direction of travel of individual cells along a computed trajectory.^45^^,^^46^ scRNAseq can be readily combined with cell surface proteomics by incorporating barcoded antibodies,^47^ and analytical techniques have been developed to infer cell-cell interactions using databases of receptor-ligand pairs.^48^

scRNAseq techniques have been widely used to study MPN, as these diseases provide an exemplar model of a cancer involving complex interactions among malignant cells, diverse immune cell types (clonal and nonclonal), and mesenchymal stromal cells. In chronic myeloid leukemia, stem cells were studied from patients before and after tyrosine kinase inhibitor treatment and from the same patient before and after transformation from chronic phase to blast crisis.^49^ Studying cells individually provided the necessary resolution to detect the rare, highly quiescent, BCR-ABL + stem cells in those responding to tyrosine kinase inhibition, and to demonstrate that these cells were transcriptionally distinct from normal stem cells, suggesting possible new targets for therapy. In this study, scRNAseq was combined with a novel method enabling BCR-ABL positive and negative cells to be reliably distinguished with high sensitivity, revealing that BCR-ABL negative stem cells also showed an aberrant transcriptional signature with activation of inflammatory pathways, especially in those patients who failed to achieve an optimal response to treatment.^49^

scRNAseq has also been applied to study how mutations alter hematopoiesis in Philadelphia-negative MPN, both in mouse models and in primary patient samples. In studies of primary cells isolated from patients with MPN, high-throughput scRNAseq has been applied to study how hematopoiesis is altered in patients with MPN.^50^^,^^51^ In a study of approximately 40,000 cells from patients with mutCALR-driven MPN, scRNAseq using a widely used droplet-based 3′ scRNAseq platform (10x Genomics, Pleasanton, CA) with a new genotyping method involving targeted amplification of mutation transcripts was performed.^50^ This method can easily be applied to profile 10 to 100s of 1000s of cells in parallel, and is sensitive for mutations that are highly expressed, such as mutCALR, for which approximately 90% cells were accurately genotyped, albeit less sensitive for low-expressed mutations (eg, 7.3% of JAK2V617F-mutant cells were genotyped) or those distant from the 3′ end, for example, SF3B1 (∼24% sensitive).^50^ This study showed that CALR mutations in patients with ET affect the entire hematopoietic hierarchy, with mutant cells detected in all stem and progenitor subsets, although a higher proportion of mutant cells in the megakaryocyte progenitor (MKP) compartment. Trajectory analyses indicated that hematopoiesis was biased toward myeloid and myeloid-megakaryocytic differentiation, with MKP showing increased cell cycling, and mutant cells had upregulation of genes involved in the unfolded protein response to an NF-kB signaling pathway.^50^ A highly-sensitive method combining genomic DNA and complementary DNA genotyping in parallel with scRNAseq was also developed in MPN, enabling resolution of transcriptional signatures of genetic subclones in MPN and confirming that nonclonal stem/progenitors show aberrant, inflammatory gene expression signatures, highlighting the importance cell-extrinsic effects of MPN mutant clones.^30^

A recent study of a mouse model of mutant calreticulin (CALR)-driven ET reported similar findings. In this model, mutCALR resulted in an expansion of both HSCs and megakaryocyte progenitors, and the investigators identified an aberrant intermediary population termed “proliferative megakaryocyte progenitors (pMKP)” that fell on a distinct differentiation pathway to MKP in normal hematopoiesis in their analyses.^52^ In addition, mutCALR HSC and MKP cell clusters showed significant dysregulation of genes involved in cholesterol biosynthesis as compared with wild-type cells,^52^ in addition to cell cycle and unfolded protein response genes as previously been described in mutCALR patients with MPN.^50^

Profiling of more than 120,000 individual cells from a range of patients with primary and secondary myelofibrosis and both *JAK2V617F* and mutCALR-driven disease also demonstrated megakaryocyte-biased hematopoiesis, with an 11-fold increase in MKP detected in patients with myelofibrosis as compared with controls in all clinical and molecular subgroups.^51^ Notably, the MKP in patients with myelofibrosis fell into 2 distinct transcriptional subgroups: a small subset with a transcriptional profile similar to MKP detected in age-matched healthy donors, and a larger population with global upregulation of inflammatory/profibrotic genes. This study also identified that megakaryocyte-associated genes, including a cell surface marker, G6B, as being widely upregulated in myelofibrosis stem/progenitor cells, suggesting a strategy for immunotherapeutic targeting of cells derived from the myelofibrosis clone.^51^

Single-cell analyses have also shed light on the changes to the nonhematopoietic stromal cell compartment in MPN. Creating a “map” of certain stromal cell populations in myelofibrosis mouse models highlighted mesenchymal progenitor cells as showing the strongest upregulation in expression of extracellular matrix proteins in fibrosis. This study highlighted the S100A8/S100A9 alarmin complex as a potential therapy target, demonstrating that its inhibition was able to ameliorate the disease phenotype in the mouse model.^53^

These insights highlight the power of single-cell “omic” techniques to accurately dissect cellular and molecular perturbations in MPN. Combined with emerging techniques to capture cell states and transcriptomes in unperturbed tissues, so-called “in situ sequencing” (see Fig. 1) single-cell approaches will prove to be a powerful approach for target discovery and in the future look set to play a key role in clinical diagnosis through more accurate disease classification and risk stratification.

## Digital pathology

The bone marrow represents a complex, dynamic, and highly regulated tissue in which diverse cell populations lie in close proximity to an orchestrated network of extracellular (stromal) matrix, blood vasculature, and bone.^54^ This complexity is compounded by physiologic changes in response to aging, stress, and environmental factors that manifest as shifting patterns of tissue cellularity, heterogeneity, and lineage maturation.^55^ Such complex spatiotemporal relationships are increasingly recognized as important for disease initiation, progression, therapeutic response, and relapse in patients with various myeloid malignancies, including MPN.^56^ Although advances in high-resolution single-cell genomic technologies are well established in the search for new treatment strategies, advanced diagnostics, and disease monitoring in MPN, complementary approaches to decipher the important interactions among neoplastic hemopoietic cells, stromal constituents, and immune cell populations of the marrow in MPN are required.^55^^,^^57^ Recent developments in digital pathology, computer vision, and image analysis have the potential to address this imbalance and revolutionize the assessment of bone marrow tissues in MPN.

Key morphologic features relating to marrow cellularity, megakaryocyte pleomorphism/atypia, and fibrosis are firmly embedded in current MPN classification schemes.^58^^,^^59^ However, inconsistencies in the interpretation of key morphologic features may lead to inaccurate diagnosis and disease classification, with multiple studies suggesting significant intraobserver and interobserver variability among pathologists.^60^, ^61^, ^62^, ^63^ Although this appears to be partly attributable to experience and training,^64^ the subjective and qualitative nature of routine marrow biopsy reporting remains a fundamental limiting factor in any classification scheme incorporating morphology-based assessment of marrow tissue. The importance and value of more accurate and objective strategies for capturing the complexity of marrow tissue architecture in MPN extends beyond the potential for improving diagnosis and classification using current recommended criteria. As perturbations in the relationship between clonal and nonclonal hematopoietic cells and components of the marrow stem cell niche are gradually elucidated using sophisticated murine models of myeloid malignancies,^55^^,^^56^^,^^65^, ^66^, ^67^, ^68^ therapeutic strategies targeting the mediators of tumor cell survival, proliferation, and chemoresistance are beginning to emerge.^69^, ^70^, ^71^, ^72^ Translating these findings to human disease and validating novel therapies will require a concerted effort to move from the conventional, subjective, and laborious description of tissue morphologic features by pathologists to objective, quantitative, and automated descriptions of marrow constituents and their interactions.

Computational analysis of digitized images prepared from glass slide material has evolved over the past few decades and significantly accelerated in recent years with the development of sophisticated deep learning (DL) methods that emulate the structure and function of human neurons in the form of artificial neural networks.^73^^,^^74^ DL methods have found ready application in the field of pathology, with image recognition convolutional neuronal networks (CNN) increasingly adopted for computer vision tasks in histopathology and cytology.^75^, ^76^, ^77^ In contrast to common solid tumors, image analysis has seen relatively limited application to disorders of the bone marrow, with most studies describing strategies for cell identification, quantification, and the resolution of specific leukemic differential diagnoses including B-ALL and common B-cell lymphoma/leukemia subtypes.^78^, ^79^, ^80^, ^81^, ^82^, ^83^ These machine learning strategies have generally relied on morphology-based criteria to distinguish tumor cell subtypes rather than interrogate tumor cells with the intention of gaining novel insights into disease biology. However, recently machine learning has been used to correlate bone marrow aspirate morphologic features with somatic mutations in myelodysplastic syndrome, with specific morphologic profiles linked to unique clinical characteristics.^84^

**Fig. 2:**
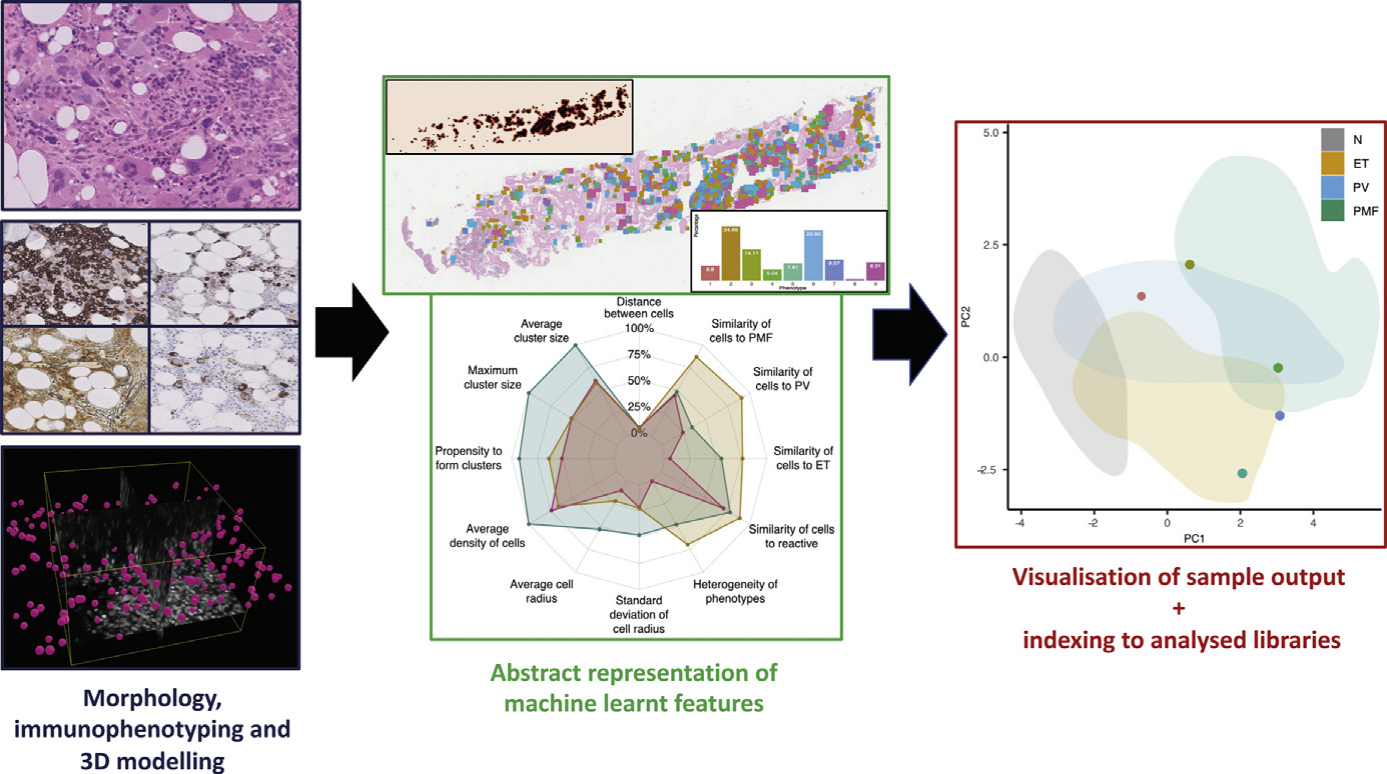
Illustration of how digital pathology approaches can be used to study cellular heterogeneity in MPN.

Despite the central role of bone marrow biopsy assessment in the diagnosis and classification of MPNs, and the importance of the marrow microenvironment in disease biology (as outlined previously), few studies have attempted to apply advanced machine learning approaches to these disorders. In response, we recently demonstrated the utility of an automated image analysis pipeline that uses machine learning techniques to extract important cytomorphological and topographic features of individual megakaryocytes from digitized images of bone marrow biopsies.^85^ This enabled the differentiation of reactive samples from common MPN subtypes (ET, PV, and primary myelofibrosis) and assisted in disease classification. Clustering of megakaryocytes using the machine-learned features from extracted megakaryocytes identified cellular subtypes beyond the sensitivity of detection by specialist hematopathologists and were seen to correlate with the underlying MPN driver mutation status. When combined with topographic assessment incorporating patterns of megakaryocyte clustering and cell distribution, the extracted features could be combined to produce a multidimensional representation of an individual sample well beyond conventional microscopic assessment. Moreover, the rapid automated analysis of samples allowed index cases of MPN or reactive marrow to be contextualized against libraries of previously analyzed samples (Fig. 2). This could be used to monitor or track morphologic features over time, corresponding to either stable disease or progression. This work highlights the potential of image analysis, driven by advanced machine learning approaches, to improve tissue diagnosis in MPN and correlate tissue-based morphologic features with standard mutational and clinical data collected during the routine investigation of patients with MPN. Importantly, the automated extraction of objective quantitative data from routinely prepared hematoxylin-eosin–stained slides is ideally suited to future integration with the results of whole-tissue immunolabeling studies, advanced single-cell genomic analysis, and the outputs from high-resolution multiplexed tissue imaging performed in the research setting.

An important consideration in the development of improved descriptions of tissue morphology and the marrow microenvironment using digital pathology and machine learning is the development of intuitive yet sufficiently detailed data visualization. The methods used will depend on the application and requirements of the end user, but it seems likely that for clinicians and researchers unfamiliar with the normal bone marrow tissue architecture and its heterogeneity, visual representation of the cellular target(s) of interest in the context of normal and/or previously analyzed disease tissue will aid interpretation and understanding. Successfully designed outputs should capture temporal changes in the course of disease or following treatment, but also have the potential to highlight relevant diagnostic and prognostic features with attention drawn to potential therapeutic targets.

Notwithstanding the potential clinical application of automated analysis of discrete single-cell populations, such as megakaryocytes in biopsies of MPN, a deeper understanding of the complex cellular interactions within the bone marrow of patients with myeloid malignancies requires a more comprehensive description of the spatiotemporal relationships that exist between the cellular and stromal components of the marrow. This will require integration of discrete cellular and extracellular morphologic features with lineage specific markers of differentiation and maturation using multiplexed immunolabeling approaches. Although several analytical platforms already use such approaches in the research setting, they are typically restricted to relatively small tissue fields (roughly equivalent to conventional microscopic high-power fields) from limited sample numbers.^86^, ^87^, ^88^ Translating the insights of such studies to cohorts of routinely prepared clinical bone marrow samples will require the development of powerful and robust computational approaches that can be adapted to identify, quantitate, and integrate diverse cellular and extracellular targets at scale. An additional challenge will be building advanced 3-dimensional models of the bone marrow environment and establishing methods for their validation using cohorts of patient samples analyzed in 2 dimensions.

Although automated analytical pipelines using convolutional neural networks to detect and segment targets of interest in digital images offer the potential for rapid sample analysis, their generation is typically dependent on access to large numbers of tissue samples accompanied by detailed clinical, laboratory, and genomic data. In general, machine learning applications in pathology use supervised approaches in which functions are learned by mapping annotated tissue features into some qualitative or quantitative output.^77^^,^^89^^,^^90^ This process is dependent on access to high-quality training data that are sufficiently labeled to allow the training phase to ultimately emulate the expert’s input data. Strategies to reduce the burden of manual annotations by pathologists include transfer learning from preexisting CNNs and the development of human-in-the-loop annotation approaches that leverage human interactions to more rapidly train, test, and validate machine-learned functions.

Given the importance of accessing sufficient quantities of high-quality training and validation material, important practical considerations surround access to suitable tissue. Few centers can rely solely on locally retained tissue archives to build and validate models of discrete MPN subtypes that are sufficiently enriched with relatively rare samples corresponding to important clinical or genomic events, such as disease transformation or response to novel therapeutics. Access to trial sample cohorts and sharing of tissue libraries between collaborating clinical centers will likely optimize use of available diagnostic material and accelerate the development and validation of machine learning models of MPN.

In summary, machine learning approaches to image analysis have already received broad acceptance in several branches of solid tissue pathology and are widely accepted as a transformational technology with significant clinical and research potential. Realizing this potential in MPN will depend on the identification and extraction of important cell-cell and cell-stroma interactions that complement and enhance our understanding of the dynamics underlying the clonal expansion of single-cell precursors that ultimately drive the disease phenotype in individual patients. This will require close collaboration among hematologists, pathologists, bioinformaticians, biomedical engineers, and software engineers and the integration of multimodality approaches spanning novel single-cell and whole-tissue sample technologies.

## Summary

Single-cell technologies have over many years provided remarkable insights into MPN biology, making conceptual advances of broader relevance across cancer biology. Future technological developments in digital pathology, in situ sequencing, and single-cell multiomics approaches (see Fig. 1) will undoubtedly be applied over the coming years to tackle crucial questions in the field. One key question that will definitively require single-cell approaches is why different HSC clones carrying MPN driver mutations such as *JAK2V617F* show such heterogeneity in fitness advantage. This is crucial to understand if we are to develop treatment approaches that reverse the fitness advantage to induce molecular responses and ultimately alter the natural history of MPN. Another key challenge in the field is to translate these single-cell technological developments through to direct patient benefit. Although at the present time it may seem farfetched for single-cell methodologies to be applied routinely in clinical diagnostics, this will surely become a reality over the coming years with obvious utility for improved approaches to diagnose and classify disease as well as monitor response to treatment and predict risk of disease progression.

## Financial support and sponsorship

None.
